# Feasibility of a protocol for deprescribing antihypertensive medication in older patients in Dutch general practices

**DOI:** 10.1186/s12875-022-01894-6

**Published:** 2022-11-09

**Authors:** Dimokrat Hassan, Jorie Versmissen, Karin Hek, Liset van Dijk, Patricia M. L. A. van den Bemt

**Affiliations:** 1 Department of Hospital Pharmacy, Erasmus MC, University Medical Center Rotterdam, Postbus 2040, 3000 CA Rotterdam, The Netherlands; 2 Department of Internal Medicine, Erasmus MC, University Medical Center Rotterdam, Rotterdam, The Netherlands; 3 Nivel, Netherlands Institute for Health Services Research, Utrecht, The Netherlands; 4 Unit of PharmacoTherapy, Epidemiology & Economics, Groningen Research Institute of Pharmacy, University of Groningen, Groningen, The Netherlands; 5 Department of Clinical Pharmacy and Pharmacology, University Medical Center Groningen, Groningen, The Netherlands

**Keywords:** Deprescribing, Antihypertensive Agents, General Practice, Frailty, Older patients, Adverse drug events, Cardiovascular Diseases

## Abstract

**Background:**

Older patients using antihypertensive medication may experience Adverse Drug Events (ADEs), and thus benefit from deprescribing. The lack of a practical protocol may hamper deprescribing. Therefore, we aimed to develop a deprescribing protocol, based on a review of literature, combined with a feasibility test in a small number of patients.

**Methods:**

A deprescribing protocol for general practitioners was drafted and tested in older patients using multiple antihypertensive medication in a single arm intervention. Patients were included if they were 75 years or older, were using two or more antihypertensives, had at least one ADE linked to antihypertensive medication and deprescribing was considered to be safe by their general practitioner. The primary outcome was the percentage of patients for whom one or more antihypertensive drugs were stopped or reduced in dose after 12 months of follow up while maintaining safe blood pressures. Secondary outcomes were the proportion of patients reporting no ADEs after 12 months and the number of deprescribed antihypertensives. Patient’s opinions on deprescribing and enablers and barriers for study participation were also collected.

**Results:**

Nine general practitioners included 14 patients to deprescribe antihypertensive medication using the deprescribing protocol. After 12 months antihypertensive drug use was lowered in 11 patients (79%). These patients had a mean systolic blood pressure increase of 16 mmHg and a mean diastolic blood pressure increase of 8 mmHg. Nine patients (64%) reported experiencing no ADEs anymore after twelve months. The mean number of deprescribed antihypertensives was 1.1 in all patients and 1.4 (range: 0.5 to 3.5) in patients who successfully lowered their medication.

At baseline, being able to use less medication was the most frequently mentioned enabler to participate in this study. The most frequently mentioned positive experience at the end of the study was using less medication, which was in line with the most mentioned enabler to participate in this study.

**Conclusion:**

A protocol for deprescribing antihypertensives in older patients was considered feasible, as it resulted in a substantial degree of safe deprescribing in this pilot study. Larger studies are needed to demonstrate the effect and safety of deprescribing antihypertensives in older patients.

**Supplementary Information:**

The online version contains supplementary material available at 10.1186/s12875-022-01894-6.

## Background

Antihypertensive medication is effective in the prevention of cardiovascular diseases with a high level of evidence, mostly based on studies in relatively young patients [1]. However, recent studies like HYVET [2] and SPRINT [3] have shown benefits of antihypertensive therapy in older patients (> 75 years) as well. Nevertheless, older patients are known to be more susceptible to Adverse Drug Events (ADEs) of antihypertensives, which increase the risk of hospital admissions [1, 4–7]. Therefore, guidelines helping to assess the benefit-risk balance for an individual patient are highly recommended [6, 7].

A recent Dutch study showed that almost 20% of the potentially preventable medication related hospital admissions in older patients were a result of ADEs like syncope, dizziness and falls. These ADEs were mainly associated with the use of multiple antihypertensive medications [4–6]. In older patients, because of frailty and pharmacokinetic changes, these side effects may occur more frequently or may be more serious [7–9]. Also, the benefits of antihypertensives in older patients depend on the estimated life expectancy [1, 4, 7, 10]. And last but not least, observational studies have shown an increased mortality in frail older patients using multiple antihypertensive drugs [7, 10, 11]. Therefore, when the negative effects outweigh the benefits in older patients, deprescribing antihypertensives may be appropriate. This is especially the case in older patients who use two or more antihypertensive medication and may suffer from ADEs caused by these medication, as these patients are at a higher risk for hospital admissions and negative impact on their health [1, 4–7].

However, deprescribing medication has proven to be difficult because of barriers perceived by both physicians and patients [10, 12–18]. Patient barriers are mostly of a psychological nature, such as fear of disease worsening when medication intake is stopped, and the feeling of worthlessness when deprescribing is suggested [12–18]. Barriers perceived by physicians are more diverse and are both of a psychological and practical nature. Feasibility, responsibility, patient concerns and logistic issues have been raised as barriers by physicians [12–17]. Furthermore, uncertainty around deprescribing remains an important factor [6, 7, 10, 14–19]. As the effects of deprescribing have not been well studied, physicians may feel insecure on how and at what moment to deprescribe in which patients. It is assumed that deprescribing antihypertensives may not be without risks and thus a safety and monitoring algorithm is required [6, 7, 20–22].

A protocol addressing the main barriers for deprescribing antihypertensive medication can serve as a useful tool and probably encourage deprescribing when needed. At a minimum, it can stimulate physicians to consider and discuss deprescribing with their patients. Therefore, we aimed to develop a deprescribing protocol to safely deprescribe antihypertensive medication for situations where the negative effects outweigh the possible benefits.

## Methods

### Study design

A deprescribing protocol was drafted based on a literature review. This protocol was tested for feasibility in older patients on antihypertensive medication in a single arm intervention pilot study.

### Development of the deprescribing protocol

A literature review was carried out in PubMed and Google Scholar. In addition, cardiovascular treatment guidelines from the European Society of Cardiology and the Dutch College of General Practitioners (in Dutch: NHG) were reviewed. The focus was on the topics of (dis)advantages of hypertension control in older patients and the process of deprescribing antihypertensive medication. The following (combinations of) key words were used: (Intensive) Hypertension OR blood pressure control, antihypertensive medication OR drugs, antihypertensives, adverse drug events, elderly, older patients, polypharmacy, deprescribing, withdrawal. The literature search was restricted to articles in Dutch and English.

Based on this literature review, we developed a deprescribing protocol for General Practitioners (GPs) and/or Practice Nurses (PNs). In the Netherlands, PNs are nurses working in General Practices who are specialized in a specific healthcare area, in which they support the GP by (partly) taking over responsibility for patient care. PNs participating in our study were specialized in cardiovascular risk management. For our protocol, first barriers and difficulties in the process of deprescribing antihypertensive medication were mapped. These barriers were subsequently addressed by our research team, each contributing with their own expertise. In the final version of the deprescribing protocol these barriers were addressed by answering the following four main questions:

1. Which patients should be selected for deprescribing?
2. How can selected patients be involved in the process of deprescribing and decision making?
3. Which antihypertensive medication should be selected for deprescribing?
4. What deprescribing and monitoring algorithm should be followed?

The main focus of this protocol was to safely deprescribe antihypertensive medication in patients who would benefit from deprescribing in general practices, in non-acute situations. The protocol does not cover patients in whom antihypertensives should be stopped immediately due to acute medical conditions.

The first version of the protocol was sent out for revision to the first participating GPs, resulting in the final version of the protocol (Supplement 1). Figures S1 and S2 in supplement 1 provide a schematic overview of respectively the deprescribing/monitoring plan and the safety algorithm, and were intended as the practical deprescribing guide. The other parts of the protocol were considered to provide background information.

### Pilot study

#### Setting

We approached 500 + general practices in the Netherlands. The majority of these practices (*n* = 500) were sent an invitation letter through the Nivel Healthcare Professionals Registries. The rest were approached via LinkedIn, through e-mail or in person. Inclusion took place between May 2019 and May 2020. The total duration of the follow-up was 12 months. The study was approved by the Erasmus MC medical ethical review committee (MEC-2019–0197). The CONSORT extension for pilot and feasibility trials checklist was followed for reporting the results (see Supplement 3).

#### Study population

Patients were included if they were 75 years or older, used two or more antihypertensive drugs, had a physical complaint mentioned in their electronic patient record that could be related to antihypertensive medication use as an adverse drug event (dizziness, syncope, falls, hypotension, headache), GP deemed deprescribing safe and were able and willing to give informed consent.

Patients were excluded when they had heart failure NYHA Class II or higher, recently had a myocardial infarction (MI) (< 12 months) or were primarily being treated by a cardiologist/internist. General Practitioners were also told to exclude patients with a systolic blood pressure (SysBP) above 150 mmHg, unless they thought deprescribing was safe and in favor despite higher blood pressure or the diastolic blood pressure (DiasBP) was lower than 60 mmHg. If DiasBP was < 60, the advice was to deprescribe antihypertensive medication anyway.

#### Study procedures

Before the start of the study, all participating GPs and PNs were instructed and informed about the deprescribing protocol. We also organized a non-mandatory course on shared decision making, both for GPs and PNs. In this course, we underlined the importance of shared decision making in deprescribing and instructed both the GPs and PNs how to apply shared decision making. In case GPs and/or PNs did not take part in the course, they were informed about the importance of shared decision making on multiple occasions, for example when we introduced our study to them. We informed them in what way shared decision making played a role in our study and how they could implement this. Furthermore, the process of shared decision making was also part of the deprescribing protocol.

A software application (as part of the software system NHG Doc® – ExpertDoc, Rotterdam The Netherlands) was developed based on the protocol to provide Clinical Decision Support (CDS) to GPs and PNs in selecting patients from their information system. The CDS software basically selected patients based on inclusion criteria when they visited the general practice. This CDS could only be used in specific brand of information systems, resulting in implementation in 16 general practices. The CDS software did not provide real-time alerts, and therefore the selection of patients was expanded by the possibility of a manual search query in the information systems. This also allowed GPs with different brands of information systems to participate.

When patients were eligible to participate, they were formally invited by their GP with a patient information leaflet. After obtaining informed consent from the patient, the GP together with the patient initiated the deprescribing process. In most cases, immediately after the GP decided which antihypertensive would be deprescribed the PN took over the deprescribing process, using the deprescribing protocol.

#### Primary and secondary outcomes

The primary outcome was the percentage of patients for whom one or more antihypertensives were deprescribed after 12 months, while still maintaining a blood pressure of 160 mmHg or lower. As this upper limit must be seen in relation to other patient characteristics and preferences, the final decision on successful deprescribing included blood pressures that were (slightly) higher than this upper limit, but still regarded as acceptable by the patient’s GP. Deprescribed medication was defined as medication that was completely stopped or reduced in dosage.

Secondary outcomes were the proportion of patients without ADEs after 12 months, the proportion of patients having at least one ADE less compared to inclusion, the number of deprescribed antihypertensives, patient’s opinion on deprescribing and enablers and barriers for study participation.

#### Data collection

##### Baseline

General patient characteristics, the names and number of antihypertensives actively in use, the dosages of the antihypertensives and the experienced physical complaints suspected to be ADEs were collected from the patient’s electronic medical records in the GP information system. A patient interview was used to confirm the ADEs they were experiencing up to a year before inclusion. We also asked patients about their reasons to participate in the study and the hesitations and potential concerns they experienced before participating in the study. We did this in a semi-structured way with open questions to all patients. The following open questions (translated from Dutch) were asked;

- What is the reason for you to participate in this study?
- What kind of hesitations or concerns did you experience, before starting with this study?

##### During follow-up

When the deprescribing process was initiated, the GP or PN kept a medical diary, which was updated each visit, reporting the deprescribed antihypertensives and the dose reduction, and when needed, the restart of the medication. At each visit, the GP or PN monitored and reported the patient’s blood pressure according to the validated office measurement of three readings. In some cases, as a result of COVID regulations in the Netherlands, patients had to monitor their own blood pressure, which was not part of the original study procedures. For this reason, these patients received a protocol for monitoring blood pressure at home, published by the Dutch Hypertension Society [23].

##### End of follow-up

The number of antihypertensives was collected from the medical records. The medical diaries were retrieved from the GPs and PNs, which were used to collect data on ADEs still present, hospital admissions during follow-up and other significant events that had occurred. Patients were interviewed on their opinions on deprescribing as well as their experiences with study participation (intensity/time investment, positive and negative aspects). For this purpose, all participants were asked the following open questions;

- What was your experience participating in this study?
- What did you think of the intensity of the monitoring process and the time needed for this?

When no negative points were mentioned, we specifically asked the following question:

- Did you have any negative experiences when participating in this study? If so, please elaborate.

#### Sample size and data analysis

A convenience sample of at least 10 patients was considered sufficient for the feasibility test. In order to keep this pilot study feasible for GPs we instructed them to include 1–2 patients per practice. All data were saved in Castor EDC (Castor, Amsterdam, the Netherlands), an Electronic Data Capture System. For data-analysis MS Excel, version 2016 (Microsoft Corp., Redmond, WA, USA) and IBM SPSS Statistics, version 28 (IBM Corp., Armonk, NY, USA) were used. Descriptive statistics were used for the primary and secondary outcomes. The questionnaire results were described qualitatively. We used inductive coding for this.

## Results

### Inclusions

In total, 23 general practices accepted the invitation to participate in our study of which in the end nine practices were able to include patients. Seven GPs and two PNs from four out of these nine practices followed the non-mandatory shared decision making course.

Each practice had between five and nine patients eligible for participation. A total of 15 patients gave informed consent for the study. One patient dropped out before the start of the study due to an initial blood pressure that was too high. Thus, a total of 14 patients were included who all completed the follow-up (supplemental 2).

An overview of patient characteristics at baseline can be found in Table 1. One patient had a baseline SysBP above 150 mmHg, but was included anyway as the general practitioner preferred to deprescribe because of the presence of ADEs.

**Table 1:** Baseline characteristics (*n* = 14)

**Age (years), median (IQR)**	80 (77–83)
**Sex (male), n (%)**	9 (64)
**BMI, mean (range)**	28 (22–35)
**Blood pressure (mmHg)**	
Systolic Median (range)	132 (116–157)
Diastolic Median (range)	72 (55–87)
**Antihypertensives in use, mean (range)**	3 (2–5)

### Antihypertensives deprescribed

In 11 out of 14 patients (79%), antihypertensive medication use was deprescribed or lowered during 12 months. In nine patients (64%) at least one antihypertensive medication was permanently stopped at the end of follow-up while in two patients (14%) the dose of one antihypertensive drug was halved. The mean number of deprescribed antihypertensives was 1.1 (range: 0 to 3.5) in all 14 patients and 1.4 (range: 0.5 to 3.5) in the 11 patients who successfully deprescribed antihypertensives.

### Change in blood pressure

The mean blood pressure (BP) – regardless of deprescribing – at the end of the follow-up was 144/77 mmHg (95% CI: Systolic BP (SysBP) 133–154, Diastolic BP (DiasBP) 71–85), with a SysBP range from 110 to 165 mmHg and a DiasBP range from 64 to 97 mmHg. The mean blood pressure at the end of the follow-up in patients in whom antihypertensives were deprescribed was 144/78 mmHg (95% CI: SysBP 133–154, DiasBP 71–85), with a SysBP range from 110 to 165 mmHg and a DiasBP range from 64 to 97 mmHg. This corresponded with a mean SysBP increase of 16 mmHg (range: -13 mmHg to + 44 mmHg; 95% CI: 5–29) and a mean DiasBP increase of 8 mmHg (range: -12 mmHg to + 20 mmHg; 95% CI: 1–16).

Patients who were not able to deprescribe any antihypertensive medication had a mean blood pressure of 142/75 mmHg (range; SysBP 135 to 155 mmHg, DiasBP 71 to 80 mmHg) at the end of the follow-up period.

### Adverse drug events

At 12 months follow up, 11 patients (79%) reported experiencing at least one less adverse event, of which nine patients (64%) reported experiencing no adverse events at all. Seven out of nine patients who did not experience ADEs anymore had their antihypertensive medication deprescribed. For the other two patients, cardiac issues seemed the cause of the adverse events. The adverse events in these two patients disappeared after these external factors were addressed.

Three patients (21%) reported the same number and types of ADEs at the end of the follow up. Of these patients, one used more antihypertensives, one halved the dose of one antihypertensive drug and one patient used one antihypertensive drug less compared to the start of the study. Table 2 provides a detailed overview of the outcomes per patient, compared to baseline.

**Table 2:** Outcome measures per patient, compared to baseline

Baseline			End of study		
**N**	**SysBP/DiasBP**	**ADE(s)**	**SysBP/DiasBP**	**ADE(s) still present**	**Deprescribed antihypertensives**
**1**	148/75	3 (Dizziness, nocturia, vertigo)	151/80	2 (Dizziness^c^ and Vertigo)	1 (hydrochlorothiazide)
**2**	133/72	1 (Dizziness)	137/67	0	2 (triamterene, hydrochlorothiazide)
**3**	157/87	3 (Dizziness, nocturia, headaches)	135/80	3 (Dizziness^c^, nocturia, headaches^c^)	0^d^
**4**	120/70	2 (Dizziness, headaches)	158/90	2 (Dizziness^c^, headaches^c^)	1 (chlortalidone)
**5**	135/55	2 (Dizziness, imbalance)	161/74	0	0.5 (amlodipine)
**6**	120/70	2 (Dizziness, shortness of breath)	130/85	0	1 (sotalol)
**7**	137/77	1 (Dizziness)	137/75	0^e^	0^d^
**8**	116/70	1 (Dizziness)	142/81	0	1 (lercandipine)
**9**	123/73	1 (Tiredness)	110/70	1 (Tiredness)	0.5 (metoprolol)
**10**	145/71	1 (Dizziness)	155/71	0	0^f^
**11**	139/59	1 (Imbalance)	145/75	0	1 (enalapril)
**12**	121/70	2 (Dizziness and tiredness)	165/74	1 (Dizziness^c^)	1 (amlodipine)
**13**	117/77	1 (Tiredness)	146/97	0	2.5 (barnidipine, perindopril, reduced metoprolol)
**14**	132/76	1 (Dizziness)	137/64	0	3.5 (spironolactone, hydrochlorothiazide, lercandipine, reduced losartan)
	**132/72**^a^	**1**^b^	**144/77**^a^	**0**^b^	**1**^b^

*DiasBP* Diastolic Blood Pressure, *SysBP* Systolic Blood Pressure, *ADEs* Adverse Drug Event(s)

^a^Mean

^b^Median

^c^During the deprescribing process, this ADE was temporarily not experienced by the subject

^d^Extra antihypertensive drug needed to be started instead of deprescribing

^e^Patient had mitral valve surgery, after which complaints disappeared

^f^External personal factors were the reason to restart antihypertensive medication

### Patient reported opinions on deprescribing and the study

At baseline, patients mentioned the ability to use less medication most frequently as enabler to participate in this study. Nine out of 14 patients gave this enabler to participate in the study. The second most mentioned enabler was being able to contribute to society and/or science, which was mentioned by six out of 14 patients. Furthermore, patients also mentioned being asked by their caregiver(s) (*N* = 5), improving/concerns about their health (*N* = 3), the opportunity to resolve adverse events (*N* = 2), and general interest in this topic (*N* = 2) as enablers to deprescribe their antihypertensives. Uncertainty about deprescribing and the effects on their health was mentioned by only one patient as a concerning factor or possible barrier at baseline.

At the end of the study, patients were overall very positive regarding deprescribing of their antihypertensive medication. In accordance with patient enablers to deprescribe their antihypertensives, the most frequently mentioned positive experience was using less medication, which was mentioned by half of the patients (*N* = 7). At the end of follow-up, patients also frequently mentioned having less or no adverse events as very positive (*N* = 3).

Four patients regretting either not being able to use less medication or still experiencing adverse events at the end of the study, which can be considered as negative experiences.

At the end of the study, none of the 14 patients experienced the study participation as intensive or burdening. Five of the patients even highlighted the extra monitoring and attention for their healthcare as a positive aspect of the study.

## Discussion

The deprescribing protocol for antihypertensives in general practice proved to be feasible in a small group of older patients. Antihypertensive medication was lowered in 11 out of 14 of patients, while maintaining an acceptable blood pressure. Potential ADEs were resolved in 64% of patients.

The deprescribing results are in line with the OPTIMISE Randomized controlled trial (66%), which is the only other trial that we are aware of with a similar study population and deprescribing process [19]. An important difference with our study is that the follow-up duration of the OPTIMISE trial was 12 weeks, which is considerably shorter than our study. Another deprescribing study (ECSTATIC) had a deprescribing rate of just 27% after two years in patients aged between 40 and 70 years with a low cardiovascular risk [24]. Although the reason to restart antihypertensives in patients was not always recorded for the ECSTATIC-study, available data pointed towards an increasing BP, headaches, stress and nervousness as the most likely reasons. These symptoms could explain the low success rate of deprescribing in the ECSTATIC-study [24].

The strength of our pilot study is that we have tested the deprescribing protocol in a real-world setting, which showed that our deprescribing protocol was feasible in clinical practice. Furthermore, we are not aware of other deprescribing studies exclusively including patients experiencing ADEs. Another strong enabler for patients to consider deprescribing was to use less medication, as they are using multiple antihypertensives. Both factors, patients using multiple antihypertensives and experiencing ADEs were inclusion criteria, which were shown to be strong facilitators for study participation and deprescribing.

Our study has some limitations as well. First, our study only showed the feasibility of our deprescribing protocol. The actual effect of implementing the protocol needs to be determined in larger studies, preferably using a cluster randomized design. In such studies more formal ways of data-collection need to be used, in order to guarantee data validity and limit protocol deviations. This was beyond the scope of this feasibility test. Second, although we encouraged shared decision making in more than one occasion, the course not being mandatory could have led to less optimal application of shared decision making.

Also, like many studies, our study suffered from the world-wide COVID-crisis. General practices prioritised primary care as opposed to research. Staffing issues and COVID-regulations not only negatively affected the inclusion rate, but other study aspects as well. Visits to general practices were for example restricted and thus patients had to monitor their own blood pressures, which may have resulted in less accurate measurements. In turn, this could have led to unnecessary restart of antihypertensives and thus possibly a lower deprescribing rate. However, the use of a home blood pressure measuring protocol minimized this risk of bias as much as possible. On the other hand, home monitoring by patients could increase patients’ involvement, which in turn could positively affect the deprescribing process. This should be explored in future studies, incorporating the possibilities of home monitoring into the deprescribing protocol as home monitoring may be more pragmatic than office measurements. In addition, it may increase the feeling of being in control for patients, which may further contribute to their willingness to stop medication.

Another limitation is the lack of a formal sample size calculation for this pilot study [25], which could have resulted in unforeseen problems not being detected in this pilot study. We consider this risk to be small, however, because of the extensive literature review used for our protocol and the pre-assessment of the protocol by GPs. Despite these limitations, this pilot study showed that the deprescribing protocol was feasible and safe in clinical practice. When proven effective in larger studies, the deprescribing protocol may be implemented in general practices in order to improve the quality of life of older patients on antihypertensive therapy. Future studies should look into the application of clinical decision support for deprescribing in more advanced ways than we did, and into user experience regarding such clinical decision support.

## Conclusion

A protocol for deprescribing antihypertensives in older patients was developed. The pilot study showed it was feasible to implement, resulting in a substantial percentage of deprescribing while maintaining acceptable blood pressure according to treating physicians. Larger studies are needed to demonstrate the effect and safety of deprescribing antihypertensives in older patients, preferably also taking quality of life into account.

## Supplementary Information

**Additional file 1.**

## Data Availability

The original patients’ data files will be stored for a period of 15 years. Data will also be kept in a secured electronic clinical trial database (Castor EDC) and are only accessible for members with permission. At the end of the follow-up, data in Castor EDC was verified for consistency and accurateness by an independent monitor according to Good Clinical Practice regulations. The original datasets generated and/or analyzed during the current study are not publicly available due to the risk of compromising patients privacy, but are available from the corresponding author on reasonable request.

## Abbreviations

(p)ADEs: (Potential) Adverse Drug Events
CDS: Clinical Decision Support
DiasBP: Diastolic Blood Pressure
GPs: General Practitioner
PNs: Practice Nurses
SysBP: Systolic Blood Pressure
