# Supplementary material for: Feasibility of a protocol for deprescribing antihypertensive medication in older patients in Dutch general practices
Source: BMC Prim Care. 2022 Nov 9;23:280. doi: 10.1186/s12875-022-01894-6 (PMC9644553; doi:10.1186/s12875-022-01894-6)
Supplement: Supplementary file 1 — Additional file 1. [file 12875_2022_1894_MOESM1_ESM.docx]

Supplementary content

**Dimokrat Hassan, Jorie Versmissen, Karin Hek, Liset van Dijk, Patricia M.L.A van den Bemt.** Feasibility of a protocol for deprescribing antihypertensive medication in older patients in Dutch general practices

[Supplemental 1 2](#_Toc111023940)

[Proposed protocol for safely deprescribing antihypertensive medication 2](#_Toc111023941)

[Supplemental 2 8](#_Toc111023942)

[Figure S3: Flow diagram of inclusion of general practices and patients. 8](#_Toc111023943)

[Supplemental 3 9](#_Toc111023944)

[CONSORT 2010 checklist when reporting a pilot or feasibility trial 9](#_Toc111023945)

[Supplemental 4 13](#_Toc111023946)

[Reference list of supplemental 13](#_Toc111023947)

# Supplemental 1

## Proposed protocol for safely deprescribing antihypertensive medication

**1: Which patients should be selected for deprescribing?**

Patient selection is the first step in this protocol, as for physicians or Practice Nurses (PNs) it is not always clear which patients should be approached for deprescribing antihypertensive medication.

As there are not many studies available on the effects of deprescribing of antihypertensive medication, we have made a cautious approach in which patients to deprescribe. It should be clear that only patients who arguably will benefit from deprescribing should be selected. In this protocol, criteria are included which if met, deprescribing antihypertensive medication is considered to be beneficial for these patients. These criteria are as follow:

- Patient is 75 years or older
- Patient is prescribed two or more antihypertensive medication per day
- Physical complaint(s) mentioned in patient record that, with a high probability*, are caused by antihypertensive medication use (dizziness, syncope, falls, hypotension, headache).

Age and the use of multiple antihypertensives have been associated with increased risk for adverse drug events caused by antihypertensives [1-4]

Patients should be excluded when they have one or more of the following exclusion criteria;

- heart failure NYHA Class II or higher;
- systolic blood pressure (SysBP) above 150 mmHg [1-3], unless the diastolic blood pressure (DiasBP) is lower than 60 mmHg or treating physician decides deprescribing is safe and in favor despite higher blood pressure. If DiasBP is < 60, the advice is to deprescribe antihypertensive medication anyway;
- myocardial infarction (MI) less than 12 months ago;
- Hypertension primarily being treated by cardiologist/internist.

*** as assessed by the treating physician.**

**2: How to include selected patients in the process of deprescribing and decision making?**

After patient selection, our advice is to actively involve patients in the deprescribing process through the shared decision making approach. First, it is important to inform patients as adequately as possible about deprescribing antihypertensives. Make sure they understand why they have been selected and share with them the criteria used for selection (see step 1). Inform patients thoroughly about the steps in the process of deprescribing. Tell them about the blood pressure and health monitoring process; what is the added value of this process and do they understand the possible extra burden and time investment?
Discuss alternative treatment options, what is possible for this patient in this situation? However, perhaps most importantly, inform patients about the possible benefits and risks of deprescribing. In this case the possible benefits of deprescribing antihypertensive medication are [3,5]:

- Smaller chance of potential Adverse Drug Events (ADEs) related to too intensive blood pressure regulation;
- Less medication in use and thus a smaller drug burden;
- Smaller chance for drug-drug interaction(s);
- Possible lower medication costs.

The possible risks of deprescribing antihypertensive medication given in literature are [6-8]:

- Reflex tachycardia and/or hypertension [6-8];
- Headache complaints and ankle edema [6];
- Worsening/Manifestation of heart failure [7,8];
- Changes in kidney function [6];
- Changes in heart rate [6-8];

It is also important to bring some nuance to these risks, as it may not be clear for patients how serious the risks are or how likely they will occur. The risks mentioned above seem very serious, but the chances of them happening are very small and can be further minimized by monitoring the patient (see step 4).

When the patient is well informed, figure out what is important for the patient and what his/her specific wishes are. It is important that these wishes are not assumed for the patients, but come directly from themselves. Thereafter, the pros and cons and patient’s wishes can be compared and considered in the final decision making. Be aware to let the patients have an active say if they want to deprescribe any medication or not.

**3: Which antihypertensive medication should be selected for deprescribing?**

When the decision has been made, in consultation with the patient, to deprescribe an antihypertensive drug, the next step is to figure out which antihypertensive drug should be deprescribed. The most obvious choice is to deprescribe the antihypertensive drug which is the most likely cause of the ADE(s). Unfortunately, in most cases the direct cause of the ADE is unknown. Especially the ADEs, syncope, dizziness and falls are more of a group effect and are hard to pinpoint to one specific drug. In order to select the best antihypertensive medication to deprescribe, comorbidities and practical options should be taken into consideration.

**Comorbidities**

Comorbidities play an important role in the decision what to deprescribe. Some antihypertensive medication are not only used for hypertension control, but are used in the treatment of a comorbidity as well. An example is the use of ACE-inhibitors in patients who suffer from both CVD and Diabetes Mellitus (DM). In such cases, it would be preferable to deprescribe another antihypertensive drug if possible. For a full overview of treatment of both high hypertension and other comorbidities which should be taken into account, see **table S1**.

**Table S1: Overview of conditions in which deprescribing of antihypertensive drug may be harmful due to positive effects on comorbidities**

| **Condition** | **Antihypertensives (don’t deprescribe this drug)** |
| --- | --- |
| **Asymptomatic organ damage** | |
| Albuminuria (> 3mg/mmol albumine/mmol creatinine) or >30mg albuminuria/24 hour | ACE-I, ARI |
| MI in the past | β-blocker (MI less than 12 months ago), ACE-I, ARI |
| Angina pectoris | β-blocker, calcium-channel blockers |
| Heart Failure | ACE-I, ARI, β-blocker, diuretics, spironolactone/eplerenon |
| Atrium fibrillation | β-blocker, non-dihydropyridine-calcium channel blockers, ACE-I, ARI |
| Peripheral Vascular Disease | ACE-I |
| **Other** | |
| Diabetes Mellitus | ACE-I, ARI |

**Based on the Dutch College of General Practitioners guidelines for cardiovascular risk management [1]**

ACE-I= Angiotensin Conversing Enzyme inhibitor(s); ARI = Angiotensin Receptor Inhibitor; Diuretics = thiazides ; MI = Myocardial infarction.

**Practical options**

When a patient does not have any of the above comorbidities and/or conditions, there is no specific preference for any of the antihypertensive medication [1,2]. In that case, it is recommended to take the practical side into account. The following characteristics should be considered:

- Keep long-acting antihypertensive medication as much as possible (long half-life, extended release formulations, low dosing frequencies) to promote drug adherence
- Keep in mind available/existing dosages for deprescribing. Older patients have difficulties breaking pills, for whenever they are asked to half the dose.
- Keep in mind patient’s experience. Which drug is the patient using the longest (without adverse events)? What preferences does the patient have?

**4: What deprescribing and monitoring plan should be followed?**

When an antihypertensive medication is selected for deprescribing, it is recommended to gradually deprescribe this drug and keep the patient under regular monitoring, especially in the initial phase. We have developed a strategy both for deprescribing medication as for monitoring the patients’ health during this deprescribing process. The monitoring is important and recommended to catch possible adverse events and (extreme) rising of blood pressure due to deprescribing pressure. The deprescribing strategy for the most commonly used antihypertensive medication in the Dutch GP offices is given in **Table S2.**

**Table S2.: Strategy for deprescribing antihypertensive medication in weekly intervals.**

| Antihypertensives | | | Strategy (mg/week) | | | | | | | |
| --- | --- | --- | --- | --- | --- | --- | --- | --- | --- | --- |
|  | | | **Highest daily dose in mg** | | **Following dose in mg 🡪** | | | | | |
|  | | | Week x (0) | | Week x +1 | | Week x +2 | | Week x + 3 | |
| Diuretics | | |  | |  | | |  | |  |
| - Hydrochlorothiazide | | | 25 🡪 | | 12,5 🡪 | | | 0🡪 | |  |
| - Chlortalidone | | | 25 🡪 | | 12,5 🡪 | | | 0 | |  |
| Calcium-channel blockers |  |  | |  | |  |  |  |  |  |
| - Nifedipine | | | 120 🡪 | | 60 🡪 | | | 30 🡪 | | 0 |
| - Amlodipine | | | 10 🡪 | | 5 🡪 | | | 0 | |  |
| Beta blockers | | |  | |  | | |  | |  |
| - Metoprolol | | | 100 🡪 | | 50 🡪 | | | 25 🡪 | | 0 |
| ACE-inhibitors | | |  | |  | | |  | |  |
| - Enalapril | | | 40 🡪 | | 20 🡪 | | | 10 🡪 | | 0 |
| - Lisinopril | | | 40 🡪 | | 20 🡪 | | | 10 🡪 | | 0 |
| - Perindopril | | | 8 🡪 | | 4 🡪 | | | 2🡪 | | 0 |
| - Ramipril | | | 10 🡪 | | 5 🡪 | | | 2,5 🡪 | | 0 |
| Angiotensin-II inhibitors | | |  | |  | | |  | |  |
| - Losartan | | | 100 🡪 | | 50 🡪 | | | 25 🡪 | | 0 |
| - Valsartan | | | 160 🡪 | | 80 🡪 | | | 40 🡪 | | 0 |
| - Telmisartan | | | 80 🡪 | | 40 🡪 | | | 20 🡪 | | 0 |
| Alpha blockers | | |  | |  | | |  | |  |
| - Doxazosine | | | 8 🡪 | | 4 🡪 | | | 0 | |  |
|  | | |  | |  | | |  | |  |

**Based on Nelson et al. and Luymes et al. [8-10].**

The process starts with three consecutive office measurements of blood pressure and deciding if an antihypertensive drug can be deprescribed [1,2,4,11,12]. If that is the case, the dose of the chosen antihypertensive medication is halved every week (or ceased completely if already on the lowest dose).

A weekly interval is proposed as it is practical for both the patient and the PN, as it is not too intensive, but still short enough to intervene if needed when deprescribing leads to adverse events or reflex hypertension. Thus, these contact points should also be used to evaluate (see **figure S1**) if deprescribing can be continued safely.

**Figure S1.: Overview of weekly deprescribing and monitoring scheme.**
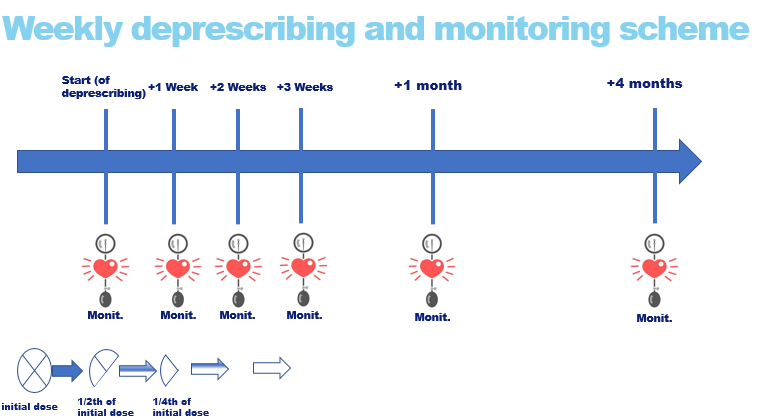
 **Monit. = monitoring**

Our safety algorithm (**figure S2**) can be used to see whether deprescribing should be continued or not. In this algorithm, we have considered the following factors;

Deprescribing should be continued, if;

- The SysBP is below 150 mmHg

AND

- The Chosen antihypertensive drug is not fully deprescribed yet *OR* ADEs are still present

The current dosage should be kept, if;

- The SysBP has risen to levels between 150-160 mmHg

OR

- The antihypertensive drug is fully deprescribed *AND* AEs are not present anymore

Deprescribing should be ceased and, if needed, the process should be reversed one step, if;

- The SysBP has risen above 160 mmHg

OR

- One or more (adverse) events mentioned under exclusion criteria have been occurred.

**Figure S2: Flow diagram of the proposed safety algorithm in the process of deprescribing.**
**SysBP = Systolic Blood Pressure; AE(s) = Adverse Event(s)**

# Supplemental 2

## Figure S3: Flow diagram of inclusion of general practices and patients.


Gen. Prac. = General Practices

GP = General Practitioners

The red color represents inclusion of general practices (Gen. Prac.), the orange color represents General Practitioners (GPs) and the blue color represents the inclusion of patients.

# Supplemental 3

## CONSORT 2010 checklist when reporting a pilot or feasibility trial


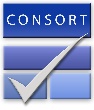
CONSORT 2010 checklist of information to include when reporting a pilot or feasibility trial*

| Section/Topic | Item No | Checklist item | Reported on page No |
| --- | --- | --- | --- |
| Title and abstract | | | |
|  | 1a | Identification as a pilot or feasibility randomised trial in the title | 1 |
|  | 1b | Structured summary of pilot trial design, methods, results, and conclusions (for specific guidance see CONSORT abstract extension for pilot trials) | 2 |
| Introduction | | | |
| Background and objectives | 2a | Scientific background and explanation of rationale for future definitive trial, and reasons for randomised pilot trial | 3 (pilot not randomised, however) |
|  | 2b | Specific objectives or research questions for pilot trial | 3 |
| Methods | | | |
| Trial design | 3a | Description of pilot trial design (such as parallel, factorial) including allocation ratio | 4 |
|  | 3b | Important changes to methods after pilot trial commencement (such as eligibility criteria), with reasons | n.a. |
| Participants | 4a | Eligibility criteria for participants | 5 |
|  | 4b | Settings and locations where the data were collected | 5 |
|  | 4c | How participants were identified and consented | 5 |
| Interventions | 5 | The interventions for each group with sufficient details to allow replication, including how and when they were actually administered | 5 |
| Outcomes | 6a | Completely defined prespecified assessments or measurements to address each pilot trial objective specified in 2b, including how and when they were assessed | 6 |
|  | 6b | Any changes to pilot trial assessments or measurements after the pilot trial commenced, with reasons | 6 (home monitoring) |
|  | 6c | If applicable, prespecified criteria used to judge whether, or how, to proceed with future definitive trial | n.a. |
| Sample size | 7a | Rationale for numbers in the pilot trial | 7 |
|  | 7b | When applicable, explanation of any interim analyses and stopping guidelines | n.a. |
| Randomisation: |  |  |  |
| Sequence  generation | 8a | Method used to generate the random allocation sequence | n.a. |
|  | 8b | Type of randomisation(s); details of any restriction (such as blocking and block size) | n.a. |
| Allocation  concealment  mechanism | 9 | Mechanism used to implement the random allocation sequence (such as sequentially numbered containers), describing any steps taken to conceal the sequence until interventions were assigned | n.a. |
| Implementation | 10 | Who generated the random allocation sequence, who enrolled participants, and who assigned participants to interventions | n.a. |
| Blinding | 11a | If done, who was blinded after assignment to interventions (for example, participants, care providers, those assessing outcomes) and how | n.a. |
|  | 11b | If relevant, description of the similarity of interventions | n.a. |
| Statistical methods | 12 | Methods used to address each pilot trial objective whether qualitative or quantitative | 7 |
| Results | | | |
| Participant flow (a diagram is strongly recommended) | 13a | For each group, the numbers of participants who were approached and/or assessed for eligibility, randomly assigned, received intended treatment, and were assessed for each objective | 8 |
|  | 13b | For each group, losses and exclusions after randomisation, together with reasons | 8 |
| Recruitment | 14a | Dates defining the periods of recruitment and follow-up | 5 (in methods) |
|  | 14b | Why the pilot trial ended or was stopped | n.a. |
| Baseline data | 15 | A table showing baseline demographic and clinical characteristics for each group | Table 1 |
| Numbers analysed | 16 | For each objective, number of participants (denominator) included in each analysis. If relevant, these numbers  should be by randomised group | Table 2 |
| Outcomes and estimation | 17 | For each objective, results including expressions of uncertainty (such as 95% confidence interval) for any  estimates. If relevant, these results should be by randomised group | n.a.; descriptive analysis |
| Ancillary analyses | 18 | Results of any other analyses performed that could be used to inform the future definitive trial | n.a. |
| Harms | 19 | All important harms or unintended effects in each group (for specific guidance see CONSORT for harms) | Table 2 |
|  | 19a | If relevant, other important unintended consequences | n.a. |
| Discussion | | | |
| Limitations | 20 | Pilot trial limitations, addressing sources of potential bias and remaining uncertainty about feasibility | 11 |
| Generalisability | 21 | Generalisability (applicability) of pilot trial methods and findings to future definitive trial and other studies | 11 |
| Interpretation | 22 | Interpretation consistent with pilot trial objectives and findings, balancing potential benefits and harms, and  considering other relevant evidence | 11 |
|  | 22a | Implications for progression from pilot to future definitive trial, including any proposed amendments | 11 |
| Other information | | |  |
| Registration | 23 | Registration number for pilot trial and name of trial registry | n.a. |
| Protocol | 24 | Where the pilot trial protocol can be accessed, if available | n.a. |
| Funding | 25 | Sources of funding and other support (such as supply of drugs), role of funders | 14 |
|  | 26 | Ethical approval or approval by research review committee, confirmed with reference number | 5 (methods; Setting) |

Citation: Eldridge SM, Chan CL, Campbell MJ, Bond CM, Hopewell S, Thabane L, et al. CONSORT 2010 statement: extension to randomised pilot and feasibility trials. BMJ. 2016;355.

*We strongly recommend reading this statement in conjunction with the CONSORT 2010, extension to randomised pilot and feasibility trials, Explanation and Elaboration for important clarifications on all the items. If relevant, we also recommend reading CONSORT extensions for cluster randomised trials, non-inferiority and equivalence trials, non-pharmacological treatments, herbal interventions, and pragmatic trials. Additional extensions are forthcoming: for those and for up to date references relevant to this checklist, see [www.consort-statement.org](http://www.consort-statement.org).

# Supplemental 4

## Reference list of supplemental

1. Nederlands Huisartsen Genootschap (NHG), 2019. Cardiovascular Risk Management (M84) [In Dutch]. NHG-guidelines. [online] Nederlands Huisartsen Genootschap (NHG). Retrieved from: [https://richtlijnen.nhg.org/standaarden/cardiovasculair-risicomanagement#volledige-tekst-medicamenteuze-behandeling](https://richtlijnen.nhg.org/standaarden/cardiovasculair-risicomanagement#volledige-tekst-medicamenteuze-behandeling)

2. Visseren FLJ, Mach F, Smulders YM, Carballo D, Koskinas KC, Bäck M, Benetos A, Biffi A, Boavida JM, Capodanno D, Cosyns B, Crawford C, Davos CH, Desormais I, Di Angelantonio E, Franco OH, Halvorsen S, Hobbs FDR, Hollander M, Jankowska EA, Michal M, Sacco S, Sattar N, Tokgozoglu L, Tonstad S, Tsioufis KP, van Dis I, van Gelder IC, Wanner C, Williams B; ESC National Cardiac Societies; ESC Scientific Document Group. 2021 ESC Guidelines on cardiovascular disease prevention in clinical practice. Eur Heart J. 2021 Sep 7;42(34):3227-3337. doi: 10.1093/eurheartj/ehab484. PMID: 34458905.

3. Lghoul-Oulad Saïd F, Hek K, Flinterman LE, et al. Prevalence and incidence rate of hospital admissions related to medication between 2008 and 2013 in The Netherlands. Pharmacoepidemiol Drug Saf. 2020;29: 1659–1668. <https://doi.org/10.1002/pds.5122>

4. Sheppard JP, Benetos A, McManus RJ. Antihypertensive Deprescribing in Older Adults: a Practical Guide. Curr Hypertens Rep. 2022 Jul 26. doi: 10.1007/s11906-022-01215-3. Epub ahead of print. PMID: 35881225.

5. Luymes, C.H. (2018, June 07). To stop or not to stop : deprescribing preventive cardiovascular medication in low-risk general practice patients. University of Leiden. Retrieved from http://hdl.handle.net/1887/63081

6. van der Wardt V, Harrison JK, Welsh T, Conroy S, Gladman J. Withdrawal of antihypertensive medication: a systematic review. J Hypertens. 2017 Sep;35(9):1742-1749. doi: 10.1097/HJH.0000000000001405. PMID: 28486271; PMCID: PMC5548513.

7. Primary Health Tasmania. (2019). A guide to deprescribing antihypertensive agents. Primary Health Tasmania (Tasmania PHN). Retrieved from <https://www.primaryhealthtas.com.au/wp-content/uploads/2018/09/A-Guide-to-Deprescribing-Antihypersensitive-Agents-2019.pdf>

8. Harrison JK, et al. Proposed antihypertensive medication withdrawal protocol. EMRAN discussion paper series 2016; issue 8.

9. Nelson MR, Reid CM, Krum H, Muir T, Ryan P, McNeil JJ. Predictors of normotension on withdrawal of antihypertensive drugs in elderly patients: prospective study in second Australian national blood pressure study cohort. BMJ. 2002 Oct 12;325(7368):815. doi: 10.1136/bmj.325.7368.815. PMID: 12376444; PMCID: PMC128950.

10. Luymes CH, Poortvliet RKE, van Geloven N, de Waal MWM, Drewes YM, Blom JW, Smidt N, Assendelft WJJ, van den Hout WB, de Ruijter W, Numans ME. Deprescribing preventive cardiovascular medication in patients with predicted low cardiovascular disease risk in general practice - the ECSTATIC study: a cluster randomised non-inferiority trial. BMC Med. 2018 Jan 11;16(1):5. doi: 10.1186/s12916-017-0988-0. PMID: 29321031; PMCID: PMC5763574.

11. Muntner P, Shimbo D, Carey RM, Charleston JB, Gaillard T, Misra S, Myers MG, Ogedegbe G, Schwartz JE, Townsend RR, Urbina EM, Viera AJ, White WB, Wright JT Jr; on behalf of the American Heart Association Council on Hypertension; Council on Cardiovascular Disease in the Young; Council on Cardiovascular and Stroke Nursing; Council on Cardiovascular Radiology and Intervention; Council on Clinical Cardiology; and Council on Quality of Care and Outcomes Research. Measurement of blood pressure in humans: a scientific statement from the American Heart Association. Hypertension. 2019;73:e35–e66. DOI: 10.1161/HYP.0000000000000087.

12. Burkard T, Mayr M, Winterhalder C, Leonardi L, Eckstein J, Vischer AS. Reliability of single office blood pressure measurements. Heart. 2018 Jul;104(14):1173-1179. doi: 10.1136/heartjnl-2017-312523. Epub 2018 Mar 12. PMID: 29530989.
